# Through health workers’ eyes: a qualitative study of health service provision for migrants at Schengen border

**DOI:** 10.1186/s12939-019-1022-2

**Published:** 2019-07-29

**Authors:** Mateja Žagar, Danica Rotar Pavlič, Igor Švab, Alem Maksuti, Boris Ilić, Martina Smrekar, Irena Kovačević

**Affiliations:** 1 Medical faculty, Department of family medicine, Poljanski nasip 58, 1000 Ljubljana, Slovenia; 2 University of Applied Health Sciences Zagreb, Mlinarska cesta 38, 10 000 Zagreb, Croatia

**Keywords:** Migrants, Health workers, Cultural diversity, Content analysis, Qualitative study

## Abstract

**Background:**

Croatia and Slovenia were the transit countries on the Balkan route for migrants and refugees from Middle East countries in 2015 and 2016. They had to optimize health care delivery in the special circumstances in refugee camps and transit centres. Little is known about health care provision in border camps where a large number of migrants stay for only couple of hours. Previous studies emphasize that language barriers and cultural differences play a central part in the relationship between health workers and migrants inside the transit zone. The aim of the study was to identify specific characteristics of health care provision experienced by primary healthcare providers in order to prepare solutions on how to organise health care in refugee settings.

**Methods:**

Twelve thematic interviews were conducted in the middle of the most intense migration movements to the North-West Europe between November and December 2015 with health workers from Croatia and Slovenia. Interview transcripts were read, coded, reviewed, and labelled. We used qualitative content analysis.

**Results:**

Four themes about the health service provision for refugees at Schengen border were identified. The circumstance when mutual understanding is poor and the consultation not successful, cultural differences represent a central barrier. Participants highlighted that the importance of respecting human dignity is crucial for the provision of basic medical care for migrants in transit.

**Conclusion:**

Successful overcoming language barriers, respecting cultural differences, humanity, susceptibility to social deprivation and traumatic experiences are the key factors important for organisation of health care in transit centers and camps. This article gives some useful tips for healthcare workers and policy makers who are participating in health services provision for migrants and other refugees. Health workers should be prepared to work in special working conditions with a lack of resources. Their work would require timely planning and reflection on the organization of more transit camps.

**Trial registration:**

Ethical Committee of the Republic of Slovenia approved the study as a project number 112/02/16.

## Background

During the immigrant crisis (15th September 2015 – 8th March 2016) 650 thousands of migrants passed through Croatia [1]. Slovenia as one of the transit countries counted more than 62000 migrant transits in January 2016 [2]. The increased number of migrants and diversity of population put pressure on health systems in Europe, especially in the provision of accessible, equitable and good quality health services [3, 4].

Countries like Norway, Sweden and many other final destinations for asylum seekers had been dealing with problems regarding the provision of quality healthcare for several years before the actual migration flows. Studies conducted between general practitioners [5–7] showed achievement of mutual understanding with migrant patients required additional efforts by health workers. They mostly had to undertake an individual approach (each patient is treated as a unique or *sui generis* case), because of the differences in their characteristics, educational level, urban or rural upbringing etc. When mutual understanding was poor, cultural differences played a central part. A Norwegian study [8] found that information about the organisation of the healthcare system and its accessibility is an important factor [9]. A study by Priebe et al. [3] underlined the importance of organisational flexibility, good service of interpreters, and the implementation of educational programmes for health workers. A study in the United Kingdom emphasized difficulties in access to health care for those refugees who don’t have support from their family and friends. Professional interpreters may be helpful when the language barriers appear, but on the other hand they can reduce the confidentiality between a patient and a healthcare worker [10]. Numerous studies highlight that professional interpreters are more likely to bridge the gaps between healthcare workers and migrants and/or other non-English-speaking patients [5, 11]. Confidentiality and trust are particularly important in the cases when patients (mostly female) are victims of physical, sexual, and emotional violence. The problem also occurs when professional interpreters as less respectful (e.g. religion of the patient), less friendly, less concerned for the patient as a person, and less likely to make the patient comfortable. In these cases healthcare workers’ role could be contra productive for the development of trust and a genuine relationship between the health worker and migrant patient [12].

Several studies highlight problems in healthcare provision for migrants [3–8, 10]. However, all these studies focused on migrants that had already decided to stay in the country involved or had at least been living there for more than six months. With the sealing of Hungary’s borders in September 2015, increasing numbers of migrants arrived in Croatia and Slovenia from Serbia. Transit and reception centres began to be established at multiple entries, transit and exit points. Emergency shelters, establishments of collective shelters in existing buildings or in tents and adaptation of buildings and sites were established to allow basic services and facilities to be provided in areas of transit [13]. Living conditions in transit camps, where they often spend months and even years, often fall well short of basic humanitarian standards because of poor sanitation, overcrowding, and insecurity [14]. Available facilities at these crossing points were put to temporary use as registration points and accommodation, but conditions were very basic, providing only protection against the elements, food distribution and emergency medical services [13]. Croatia, Slovenia and other transit countries on the Balkan route had to take a completely innovative approach to provide good healthcare for migrants, because they were passing through and staying in one place for only a couple of hours. The migration flow through Croatia and Slovenia was enormous. The number of migrants who were accepted and provided in some centres significantly exceeded the planned number and their capabilities [15].

Illegal border crossings, cold weather (November and December 2015), lack of resources in reception centres etc., further complicated the situation. Acute medical care was provided for migrants who needed help, especially vulnerable groups. Responding quickly and efficiently to the arrival of large groups of people from abroad requires effective coordination and collaboration between and within countries as well as between sectors. A good response to the challenges faced by migrant groups requires good preparedness. High-quality care for refugee and migrant groups cannot be ensured by health systems alone [16]. The experience of improvised primary care settings as globalised and culturally neutral unites in order to ease the connectivity between patients and health workers in the transit zones were reported from the border site [17].

This study examined the views and experiences of healthcare professionals on the health care of migrants at border camps during the migrant crisis. We conducted interviews directly after mass movements, when the media warned of many health risks both of migrants and of local residents. Previous studies emphasize the problems of the daily provision of adequate health care to migrants in clinics in primary care. Little is known about health care provision in temporary border camps with different circumstances and large group of migrants. We thought that we would find poor quality health care and bad responses due to chaotic conditions. We wanted to explore the experiences of healthcare professionals and organization of health care in the border camp, main weaknesses at providing health care and the relationship between migrants and health care professionals. The idea of the research was created when researchers DRP and MŽ visited the camp on the field (Šentilj). Researchers’ preconceptions were that may be too stressful for participants to talk about some events. Some were also emotionally disturbed and affected. Researchers’ methods of opening up and focusing on interviewees’ interaction were to establish trust, respect and openness for participants. In order to achieve this, the participants voluntarily chose if they wanted to talk about their experiences with working with migrants and the interviews took place at their home environment. In a relaxed conversation in their environment and our understanding of working in a very hard conditions the interviewees could freely responded to descriptive questions. We allowed enough time and encouragement to describe their experiences supported with examples. The interviewees shared with us their experiences, disappointments and surprises. The authors decided to focus only on health care providers in border camps because of their experiences and perspectives to contribute to the knowledge on the topic and obtain many useful information about migrants health care provision in border camps. Our primary interest was to identify the problems faced by primary health workers at border camps in order to prepare suggestions on how to prepare health care in future waves of massive border crossings.

## Methods

### Participants

Sampling in the initial stages of a study involve the purposeful selection of a sample. The sample is not selected from the population based on certain variables prior to the study but the researcher starts the study with a sample where the phenomenon occurs [18]. According to that fact that the study is “in the initial stage” [18], we decided to interview Slovenian and Croatian health workers that we believed would maximize the possibilities of obtaining data about their experience with health service provisions for migrants on Schengen border. Twelve health workers from Slovenia and Croatia were included in the study on account of their willingness to participate and the answers started to repeat so saturation was achieved. When selecting the participants, we considered the following criteria: they have available the knowledge and experience that the investigators need (working with migrants on the Balkan route in November 2015); they are capable of reflection (they remember events and are not biased); they had time to be interviewed; and they were willing to take part in our study. Only health workers who voluntarily chose to speak about their experiences took part in the study. Informed consent was obtained from all individual participants included in the study. The doctors and nurses included in this study are all health professionals, specialists who otherwise work in a typical healthcare facility: family medicine clinics and / or emergency medical care, and have a lot of experience in providing health care. Demography of interviewed health workers is presented in Table 1.

**Table 1:** Demography of interviewed health workers

Health worker (HW) identification (ID)	Profession	Country of origin	Area of operation (migrant camp)	Health facility	Gender	Language skills	Reason for work
HW1	Doctor of family medicine	Slovenia	Vrhnika	Primary health care	Female	Slovenian, English, Croatian	Voluntarily
HW2	Doctor of family medicine	Croatia	Opatovac, Slavonski Brod	Primary health care	Female	Coatian, English	Voluntarily
HW3	Nurse	Croatia	Slavonski Brod	Primary health care	Female	Croatian, English	Compulsory
HW4	Nurse	Slovenia	Brežice	Primary health care	Female	Slovenian, English, Croatian	Voluntarily
HW5	Doctor of family medicine	Slovenia	Dobova	Primary health care	Male	Slovenian, English, Croatian	Voluntarily
HW6	Doctor of family medicine	Croatia	Slavonski Brod, Bjeliš	Primary health care	Male	Croatian, English	Compulsory
HW7	Doctor of family medicine	Croatia	Slavonski Brod, Opatovac	Primary health care	Female	Croatian, English	Voluntarily
HW8	Doctor of emergency medical assistance	Croatia	Koprivničko-križevačko	Emergency	Male	Croatian, English	Compulsory
HW9	Doctor of emergency medical assistance	Slovenia	Vrhnika	Emergency	Female	Slovenian, English, Croatian	Compulsory
HW10	Doctor of family medicine	Croatia	Slavonski Brod	Primary health care	Female	Croatian, English, French	Compulsory
HW11	Nurse	Croatia	Slavonski Brod	Primary health care	Female	Croatian, English	Voluntarily
HW12	Doctor of emergency medical assistance	Croatia	Harmica	Emergency	Male	Croatian, English	Compulsory

### Materials and methods

#### Study design

The study is analogue to other qualitative studies claim to describe life-worlds “from the inside out” [19], from the point of view of the participants in the health care of migrants [3, 4, 7, 20–22]. The study is based on qualitative thematic interviews with 12 health workers who were actively involved in health service provision for migrants on the Schengen border at the end of 2015. Since the interview location “plays a role in a constructing reality” [23] we allowed the interviewees to decide where the interviews will take place. All interviews were conducted in nine health facilities in Croatia and Slovenia. The data were collected between November and December 2015.

The interviews conducted were characterized by three criteria [24]: problem centring, that is, the researcher’s orientation to the relevant problem (i.e. health workers prior expectations and factual experiences with health service provision); object orientation, that is, developing or modifying interviews with respect to an object of research (i.e. health workers specifics in their outreach work); and process orientation, that is, understanding of the object of the research (normative grounding of health provisions in culturally diverse environment and dynamics and actual experiences regarding problems/barriers, working conditions etc.). The questionnaire was developed in the following manner: after studying the literature from the field “migration and health”, the first set of questions was developed. This set was then discussed among researchers in order to narrow the topics. The exact wording of the questions was developed in consultation with researchers and some health workers that were working with refugees in the past. The questionnaire covered 15 descriptive questions. Healthcare professionals described their experiences in dealing with migrants in migrant centres. Issues also related to the organization, cooperation and main obstacles in ensuring the health care of migrants. They also presented us the responses of migrants and their dilemmas and suggestions for improvement and provision with better quality health care to migrants. In interview conversations with health workers we adopted a thematic interviewing [25] approach. The authors of this study carried out interviews with health professionals. Researchers had been prepared on the topic and tried to make the most of the interviews in order to obtain as much useful information as possible for our research. Interviews were performed and recorded personally, face-to-face with health professionals. For each interview, we spent 15 to 45 min, which was largely dependent on the health worker. All the interviews were recorded [26] and transcribed verbatim. Interviews were conducted in Slovenian and Croatian language.

### Qualitative content analysis

We conducted a qualitative study using the method of qualitative content analysis [26–28]. We have used inductive content analysis that includes coding, creating categories, and abstraction – framing a general description of the research topic through generating categories [27]. We used the software *Atlas.ti* [29] open coding procedure [28] based on breaking data apart and delineating concepts to represent blocks of raw data. Two researchers independently coded the interviews, while the other researchers supervised the process. If consensus was not reached we tried to achieve intercoder agreement [30] about differently perceived parts of the analysed text to fit the created category (also known as a unitizing process) [26, 31].

Ethical Committee of the Republic of Slovenia approved the study as a project number 112/02/16.

## Results

Seventeen categories, 72 codes with a total frequency of 523 were identified. Four themes referring to Croatian and Slovenian health workers experiences with health service provision for migrants on Schengen border were formed. We named themes depending on the content they illustrate.

### Organization of health services on the ground

The first theme includes three categories related to the organization of health services on the ground: country specifics; organization of work in (transit) migrant camps; and working conditions (e.g. organization of work, handling equipment and logistics etc.).

Interviewed health workers have exposed some country specifics addressing the migrant crisis. Those specifics were related to Slovenia’s and Croatia’s unwillingness for the wave of migrants with the consequent impact on the health service provisions. The unwillingness of the countries refers to the number of migrants who were accepted in some border centers and significantly exceeded the planned number and its capabilities. In that context some of the health workers have emphasized chaotic situation (in some of the migrant camps). They mentioned the problem of the number of migrants (the trains with migrants were not predicted), the size of migrant camps, lack of human resources and basic human necessities.

This is directly connected with the organization of work and working conditions in migrant camps. Different health workers had different experiences. Some of them had witnessed good organization of work without problems with necessary equipment and logistics, while others mentioned inadequate organization and problems with medical equipment and other supplies. There were differences in the opinions of doctors and nurses regarding the organization. Despite experienced health professionals, they all found themselves in such conditions of work for the first time. The quotations below show that there were differences in positions.

*My impression is the camp as a whole functioned perfectly and was very well organized, all services. I would say everything was perfect, as far as possible (HW6, Slavonski Brod, Bjeliš);*

*In the camp health care was not adequately provided. There was something but definitely not enough for routine care standard for refugees, as we know it today (HW2, Opatovac, Slavonski Brod).*

### Problem areas

Four problem areas were identified: communication (language barriers); migrants’ social deprivation and traumatic occurrences; negative attitudes among health workers and migrants; and cultural differences. Those categories are broad and comprehensive, and they include different problems we recognized though coding interviews.

Probably the biggest and most common were communication problems. Data obtained in some of the previous studies (e.g. 3) indicated that the language barrier is the biggest obstacle for comprehensive health service provision for migrants. Our study showed that making a diagnosis, due to language difficulties, was real challenge for health workers. The latter was in permanent stress due to incomplete communication and possible wrong diagnosis or misidentified treatment of migrants that needed health service provisions.

Some interviewees outlined translators while others used different techniques to communicate with migrants. Present translators were mostly volunteers, i.e. health workers did not have a translator as an integral part of their medical team. In that context some interviewees engaged:

*Google translate and tried to pronounce some Arabic words (HW6, SlavonskiBrod, Bjeliš).*

other have tried to improvise and use:

*Arms and legs to explain something (HW10, SlavonskiBrod).*

An additional problem was migrants’ social deprivation and traumatic occurrences. Those people came from war zones and besides medical problems they survived different war situations, which resulted in social deprivation and traumatic occurrences. Migrants were therefore suspicious and introverted. Majority of interviewed health workers outlined the importance of the psychological (moral) support, understanding, and a sense of security and acceptance. Most common diseases, injuries and other problems were: malnutrition, injured foot, diarrhoea and vomiting, respiratory infections and colds. For the majority of refugees medical treatment was less important which is best illustrated by the statement of one of the interviewees:

*Migrants are mainly healthy, but exhausted (HW6, SlavonskiBrod, Bjeliš).*

Most interviewees expected migrants to be sicker than it turned out later. Most migrants were young and healthy and were capable to make long and hard way and because of that exhaustion was the most frequent problem. Children and young families were also not very ill at all. Healthcare professionals described cases of more serious ill elderly people with chronic illnesses and pregnant women. The results of social deprivation and trauma experiences were negative attitudes among health workers and migrants. The latter did not want to be separated from the group; they mostly rejected hospitalization and more detailed medical examination because of fear. Partly this could be also explained through cultural differences. Majority of migrants were Muslims from socially deprived parts of Syria, Afghanistan and Iraq. According to their cultural heritage those people sometimes have different understanding of illness and treatment, which was also mentioned by some health professionals. Some of the interviewees emphasized issues about privacy, family ties and ethical dilemmas (should they stay in the camp or should they go further; should they leave their children in a hospital etc.). All of this further hampered the work of health workers at the ground.

### Positive experiences

The third theme also includes four categories. The first category inside the third theme we have called positive attitudes among health workers and migrants. It is followed by philanthropy, medical oath and human ethics, and cultural awareness of health workers.

All of the interviewed health workers emphasized at least one positive attitude about migrants. They were friendly and grateful for the help. This has affected health workers to be even more philanthropic:

*To do their job in accordance with a medical oath and human ethic (HW2, Opatovac, Slavonski Brod).*

Cultural awareness of health workers was reported as an important element for successful actions. In that case Croatian and Slovenian medical profession deserve compliments for culturally sensitive service during the migrant crisis.

Some health workers exposed that the only problem was lack of human resources. In that context migrant crisis – from the medical point of view – was the result of inappropriate organization and disinterestedness of national governments to solve the problem more seriously, which was shown in the following statement:

*I was frightened of the organization, where will we get enough people to work with the refugees (HW9, Vrhnika).*

Interviewed health workers have shown readiness to re-engage in similar campaigns, which additionally highlights positive experiences with the (mostly) humanitarian crisis.

### Social dimension of the migrant crisis

Our fourth theme is reserved for a social dimension of the migrant crisis. This theme includes two categories (lack of food and other necessities; fear and uncertainty) that best exemplify the essence of the migrant crisis.

Beside organizational chaos, lack of human resources and inadequate working conditions in migrant camps, health workers have also emphasized the social dimension of a humanitarian crisis. According to some authors the current migrant crisis in Europe is a test of European values. In other words, the current migrant crisis in Europe “will truly prove if Europe can live up to its founding principles of human dignity, solidarity, freedom, democracy and equality” [32]. Migrants were concerned about how their problems would be solved and what would happen to them. Health care professionals observed their anxiety and thinking about the need for psychological support. Basically all interviewed health workers expressed sympathy with migrants:

*The soul hurts them, they really needed someone to treat them as human beings (HW1, Vrhnika).*

Concerns were where people go, what to expect from the future, and do they have real chances for integration in the final host country.

Based on concerns how migrants will cope with an uncertain future in a foreign country, we can say that health workers had multiple functions on the ground. Besides their medical profession they were also psychologists, social workers and philanthropists. Such positions of health workers provide them an ability to understand all dimensions of the crisis, humanitarian crisis. They tried to draw attention to the permanent shortage of food and other basic necessities (infant foods, hygiene gadgets, blankets, tents etc.). They also tried to talk about migrants’ fears and uncertain future. In that context moral support was most important.

As potential measures to mitigate the migrant crisis, interviewed health workers also made some suggestions. Based on their experience in working on the ground they suggested it is necessary to introduce a permanent emergency services, and provide permanent health care for all migrants, no matter where they are located. More sophisticated proposals were focused on the inclusion of international organizations – concretely:

*Organization Magna, which is an international organization for pediatric health (HW10, Slavonski Brod).*

## Discussion

### Main findings

Four themes (organisation, problem areas, positive experiences and social dimension) were identified in the delivery of medical care to migrants. The main problem area was the communication between health workers and migrants. Other problem areas included refugees’ social deprivation and traumatic occurrences, negative attitudes among health workers and migrants and cultural differences. The European values, such as human dignity, solidarity, freedom, democracy and equality were tested when the migration flow at the Schengen border began to increase. In the Constitution of the World Health Organization (WHO) it is defined that the enjoyment of the highest attainable standard of health is one of the fundamental rights of every human being. Access to responsive, people-centered health systems is essential to ensure available health care for all refugees, asylum seekers and migrants throughout the migration journey [33].

Our findings extend the findings from previous studies in the field [3–6, 8, 10, 17, 34], confirming that communication barrier is the biggest obstacle in the work with migrants on the ground. They also confirmed that social deprivation, traumatic experiences, cultural differences and different understandings of illness and treatment all play an important role when trying to provide optimal healthcare for migrants in transit countries. A qualitative study where healthcare workers and migrants were included emphasized that the biggest barriers in accessing healthcare for migrants were time pressure, lack of trust and information, language differences and less frequently cultural barriers. The study was conducted in seven European countries and not only in transit centres. Migrants expressed desire for compassionate, culture-competent healthcare workers, in whom they can find trust, who involve interpreters when needed [35].

The most important components of good practice to be implemented in transit countries are good organisation, sufficient and readily available interpreting services and good access to psychological support and social workers [36]. The good organisation includes providing a sufficient number of human resources, medical equipment and basic human necessities for refugees. Available interpreting services would enable better communication and could minimise discrepancies of different understandings of illness and treatment. Considering the fact that most migrants that entered the transit countries came from war areas, the majority of them suffered different traumatic events. This is why well-structured moral, psychological and social support needs to be provided in order to implement good and quality healthcare for migrants. The health workers involved however have proven to be extremely philanthropic and provided great moral support. They served not only as medical professionals but also as psychologists and social workers. Migrants were proven to be friendly and grateful for the help they got, although they sometimes rejected hospitalisation and detailed medical examination because of fear and/or in order not to be separated from their families. A study by Stammel et al. [37] emphasizes that the received multidisciplinary treatment in a clinic had a positive effect on trauma-related symptoms as well as on the quality of life of traumatized refugees where medical doctors, psychotherapists and social workers were included in the treatment process. A study by Mechili et al. [38] also emphasizes a trained multidisciplinary team in order to provide culturally adapted health assessment and compassionate health care services for migrants. For better treatment migrants must be informed about the provision of healthcare services across European countries and should stay in one host country longer.

High-quality care for refugee and migrant groups cannot be ensured by health systems alone [16]. Managing and addressing the complexity of migration is not only an issue for the health sector but for the whole government, across public policies, and local, national and regional development agendas [33]. The migration crisis in Europe demonstrated that the capacity of individual countries has been pushed to the limit and that the development of resilience to sustained migration is needed. Public health preparedness is not optimal in many countries, with improvements needed in multisectoral approaches and health systems capacity to address the health needs of large influxes of refugees, asylum seekers and migrants, including in preparedness, surveillance and response, and public health participation in health systems planning and development [33]. The fact is that national governments were not well prepared and/or did not show enough interest for the huge number of migrants that crossed the transit countries, which led to inefficient organisation and lack of human resources, medical equipment and other supplies [13, 14]. This was also felt by health professionals in their work with migrants in transit centres. Besides the context of politics in most European countries, countries at this moment are not much in favour of welcoming migrants, which limit the attention to adequate service planning and delivery [35]. Migrant crisis as a humanitarian crisis needs courageous politicians with a will to act for sufficient aid to migrants. The Authorities should be aware migrant health care provisions and the work of health workers depend on timely planning and adjusted interpretation of existing national and European Union (EU) legal framework (e.g. Schengen Borders Code). It would be reasonable to consider organizing a larger number of smaller migrant camps where health care provisions could be provided easier and much more effective. A different implementation of existing legal frameworks would provide certain benefits for health workers (e.g. smaller centres are less crowded, and it is easier to give assistance to patients), and for migrants, who need more philanthropic attention and fewer administrative barriers.

### Methodological considerations

Besides several strengths, such as thematic interviewing, consistent use of qualitative content analysis and the paucity of researches about migrant health care in overcrowded transit centres, the study also has some limitations. General limitation of the study is related to epistemological criteria and validity in qualitative research [39]. Although qualitative studies provide richness in detail, large-scale representative quantitative surveys are needed to capture a large amount of data and shed more light on health workers’ experiences with the refugee crisis. The using of mono-methods: thematic interviewing only health care workers can result in limited triangulation. Another limitation is linked to the purposive sample used in the study. Our study was focused on health workers from Slovenia and Croatia. The participants in our study were selected according to their willingness to participate. Although the number seems small, the richness of their testimonies has given us enough information to reach conclusions. We have also achieved saturation, which is a standard of quality in qualitative methodology. We are aware that the qualitative approach has its limitations [40] and know, that an additional study with random participant selection and a quantitative approach would ideally complement our findings, putting the importance of the findings in perspective.

### Impact of study

The study provides an overview of the experience of health professionals in the provision of health care for migrants in transit centres. It is different providing adequate health care for migrants who stay or live longer in the host country than in transit centres. The situation in clinics is better than delivering health care in emergency tents and facilities where chaos, overcrowding, and the lack of basic needs and human resources have been caused by a number of migrations. In addition to social deprivation, traumatic experiences, negative attitudes between the healthcare worker and the migrant, and cultural diversity, the main barrier is the language barrier. The study emphasizes the need for the better organization by the government, ensuring the continued availability of a professional interpreter, and preparedness of health professionals to work in such conditions. Good care of migrants in transit centres, a positive, trusting and respectful relationship between the healthcare worker and the migrant is urgent and the possibility of multidisciplinary treatment is essential.

## Conclusions

Findings revealed some information that may be seen as useful tips for all health care workers and policy makers who face migrant crisis and participate in health service provision for migrants and other refugees. It is necessary to provide more permanent accessible professional interpreters. Irrespective of the circumstances, and the scope of any humanitarian crisis, health workers should be aware that successful overcoming of language barriers and the observance of cultural differences are key factors for successful treatment of migrants. A useful set of skills for health care providers includes compassion, susceptibility for social deprivation and traumatic experiences of those people. It is necessary to provide more health professionals and multidisciplinary treatment, cooperation with psychologists, psychiatrists and social workers. More than professional health care migrants need to feel health workers’ humanity and willingness to help them. However, health workers should be mindful that providing assistance during crisis takes place in special working conditions with shortages of everything: adequate facilities for patients’ treatment and other medical equipment; human resources; food and other necessities; translators other actors involved in the social care of migrants (e.g. administrators; representatives of state authorities (police, army, social workers) etc.). The government could consider a better organization, an increasing number of migrant centres that would reduce overcrowding and improve the conditions for providing adequate medical care for migrants.

Our findings extend the findings from previous studies and make some contribution to the literature in the field. Our study contributes to important information in the research gap regarding the main obstacles to providing health care for migrants only in transit centres during the migration crisis, which is a real challenge for individual countries when the migration centres become overcrowded.

## Data Availability

The datasets used and analysed during the current study are available from the corresponding author on reasonable request.

## Abbreviations

EU: European Union
HW: Health worker
ID: Identification, identity
WHO: World Health Organization
